# Allium test genotoxicity data on water from river valley of Irtysh, West Siberia

**DOI:** 10.1016/j.dib.2022.107861

**Published:** 2022-01-23

**Authors:** Dmitry S. Pesnya, Sergey E. Bolotov

**Affiliations:** a Papanin Institute for Biology of Inland Waters Russian Academy of Sciences, Yaroslavl Region, Nekoyz District, 152742 Borok, Russia; b Laboratory “AquaBioSafe”, Tyumen State University, Volodarskogo st., 6, 625003 Tyumen, Russia; c Higher Environmental School, Ugra State University, Chekhova st., 16, 628011 Khanty-Mansiysk, Russia

**Keywords:** Allium test, Mutation research, Chromosomal aberrations, Micronuclei, Siberia, Irtysh river, Floodplain

## Abstract

Assessment of genotoxicity of aquatic ecosystems is of great importance for environmental management and assessment of water ecological quality as well as health safety.

Data on the genotoxicity of water in rivers of such a large region as Siberia are almost absent. Researchers do not pay enough attention to river floodplains, but such studies are important for assessing the ecological condition of the river system, because a significant part of toxic and genotoxic substances accumulates in the river floodplains

A unique feature of the Ob-Irtysh interfluve is its vast floodplain, the largest in the Northern Hemisphere, providing key ecological functions of the territory. Originality of this river system lies not only in enormous size, but also in the exceptional duration and magnitude of spring-summer floods and the formation of complex biogeocenosis.

In this data article we provide for the first time genotoxicity data for the Irtysh river valley (West Siberia) which is longest tributary river in the world. Water samples were collected from 5 sites: Irtysh river, floodplain sites (Jivaya river, Mukhrina river, Baybalak river) and terrace (Bog Mukhrino). Allium test was used to assess genotoxic effects. This method is one of the recommended bioassays for rapid genotoxicity screening of the water. Ana-telophase chromosomal aberration assay and micronuclei test were performed to determine genotoxic effects. Obtained data for mitotic index and other phase indexes. Data on water genotoxic effects are accompanied by data on physicochemical parameters. The data shows that the floodplain waters accumulate allochthonous organic matter, which is evidently supplied with high water. This determines the increased genotoxicity of floodplain waters. The data allow other researchers to conduct a comprehensive analysis of the genotoxicity of natural waters on the landscape gradient of a river valley (terrace-floodplain-river) and reveal possible causes of the observed effects.

## Specifications Table

**Table:** 

Subject	Environmental science
Specific subject area	Health, Toxicology and Mutagenesis Environmental genotoxicity
Type of data	Table Excel file Image
How the data were acquired	Cells analysed under light microscopy, 400-1000x (Cnoec microscope). Potencymetry (portable multiparameter zonde YSI Pro), Titrometry (Winkler method). Spectrophotometry (Hanna C-200 multiparameter analyzer). Cells photographed with MI camera.
Data format	Raw Analyzed
Description of data collection	The main physico-chemical parameters of waters at the sampling sites in the Irtysh river valley were obtained in the summer (August) 2021. Genotoxic effect (chromosomal aberrations, mitotic abnormalities, micronuclei, mitotic index) evaluated using root meristems of Allium cepa L. var. Shtuttgarten (Allium test bioassay).
Data source location	Papanin Institute for Biology of Inland Waters Russian Academy of Sciences, Yaroslavl Region, Nekoyz District, 152742 Borok, Russia Laboratory “AquaBioSafe”, Tyumen State University, Volodarskogo st., 6, 625003 Tyumen, Russia Higher Environmental School, Ugra State University, Chekhova st., 16, 628011 Khanty-Mansiysk, Russia GPS coordinates for collected samples within this article
Data accessibility	Pesnya, Dmitry; Болотов, Сергей (2021), “Allium test genotoxicity data on water from river valley of Irtysh, West Siberia”, Mendeley Data, V1, doi:10.17632/ygvmyjc36v.1[1] Direct link to raw data: https://data.mendeley.com/datasets/ygvmyjc36v/1

## Value of the Data

• The value of the presented data lies in the fact that it were obtained in the gradient section of the river - floodplain - terrace as part of the structure of a large Siberian river valley, which provides valuable comparative material for other studies of biological effects in river floodplains.
• The data is useful for specialists (environmental scientists, toxicologists, ecologists) who are involved into environmental research, monitoring and protection of water resources.
• Data is necessary for environmental management programs to ensure the environmental safety of the lower Irtysh water system.
• This data can be a model for comparative studies and assessment of the genotoxic effects of another floodplain river ecosystems.
• Water genotoxicity data are accompanied by physico-chemical data for a comprehensive characterization of the river water ecological quality.

## 1 Data Description

The main water physico-chemical parameters at the sampling sites of the Irtysh river valley were obtained in the summer (August) 2021 and shown in Table 1. Table 1 presents the main hydrological characteristics of water bodies (water depth and temperature), and important chemical parameters of the aquatic environment - pH, electrical conductivity, redox potential, content of dissolved oxygen, labile organic matter (BOD_5_), biogenic (nitrates and phosphates) and typomorphic (iron and aluminum) ions.

**Table 1:** Physico-chemical data of natural water at sampling points in the river valley of Irtysh, West Siberia.

Water parameter	Bog Mukhrino	Jivaya river	Mukhrina river	Baybalak river	Irtysh river
Depth, m	0.4	1.0	1.4	7.0	9.7
Temperature, ^о^С	22.1	22.8	23.3	23.9	23.5
pH	3.8	7.3	7.0	7.7	8.0
ORP, mV	353.3	212.8	201.8	219.6	174.2
Conductivity, µSm/cm	35.6	167.2	173.7	206.8	215.0
BOD_5_, mgO_2_/L	1.26	2.27	2.49	2.12	1.70
Dissolved oxygen, mg/L	5.71	5.49	6.46	6.10	6.18
NO_3_^−^, mg/Ll	0.95	0.08	0.15	0.36	1.60
PO_4_^3−^, mg/L	0.11	0.21	0.13	0.18	0.23
Al^3+^, mg/L	0.10	0.14	0.15	0.25	0.20
Fe^2+,3+^, mg/L	0.26	1.65	1.47	1.28	1.23

Calculated data for genotoxic effects (mitotic index, phase indexes, frequency of mitotic abnormalities, chromosomal aberration, micronuclei and total frequency of abnormalities) shown in Table 2.

**Table 2:** Data on genotoxic effects in the meristematic cells of *A. cepa* roots.

Parameters of genotoxic effect	Control	Bog Mukhrino	Jivaya river	Mukhrina river	Baybalak river	Irtysh river
Mitotic index, %	6.648	6.503	7.769	9.692	8.360	8.996
Chromosomal aberration, %	0.224	0.478	0.481	2.783	1.483	0.396
Total abnormalities, %	0.468	0.656	0.588	3.827	1.636	0.543
Micronuclei, ‰	0.030	0.030	0.040	0.160	0.060	0.020
Prophase index, %	45.996	45.088	46.371	43.081	48.613	46.379
Metaphase index, %	20.865	20.848	20.718	26.635	19.774	18.814
Anaphase index, %	16.930	14.674	14.658	9.067	10.842	16.068
Telophase index, %	16.208	19.390	18.253	21.217	20.771	18.739

The raw and calculated data are provided in an Excel file available in the Mendeley data repository which can be found with the title “Allium test genotoxicity data on water from river valley of Irtysh, West Siberia”.

**Fig 1:**
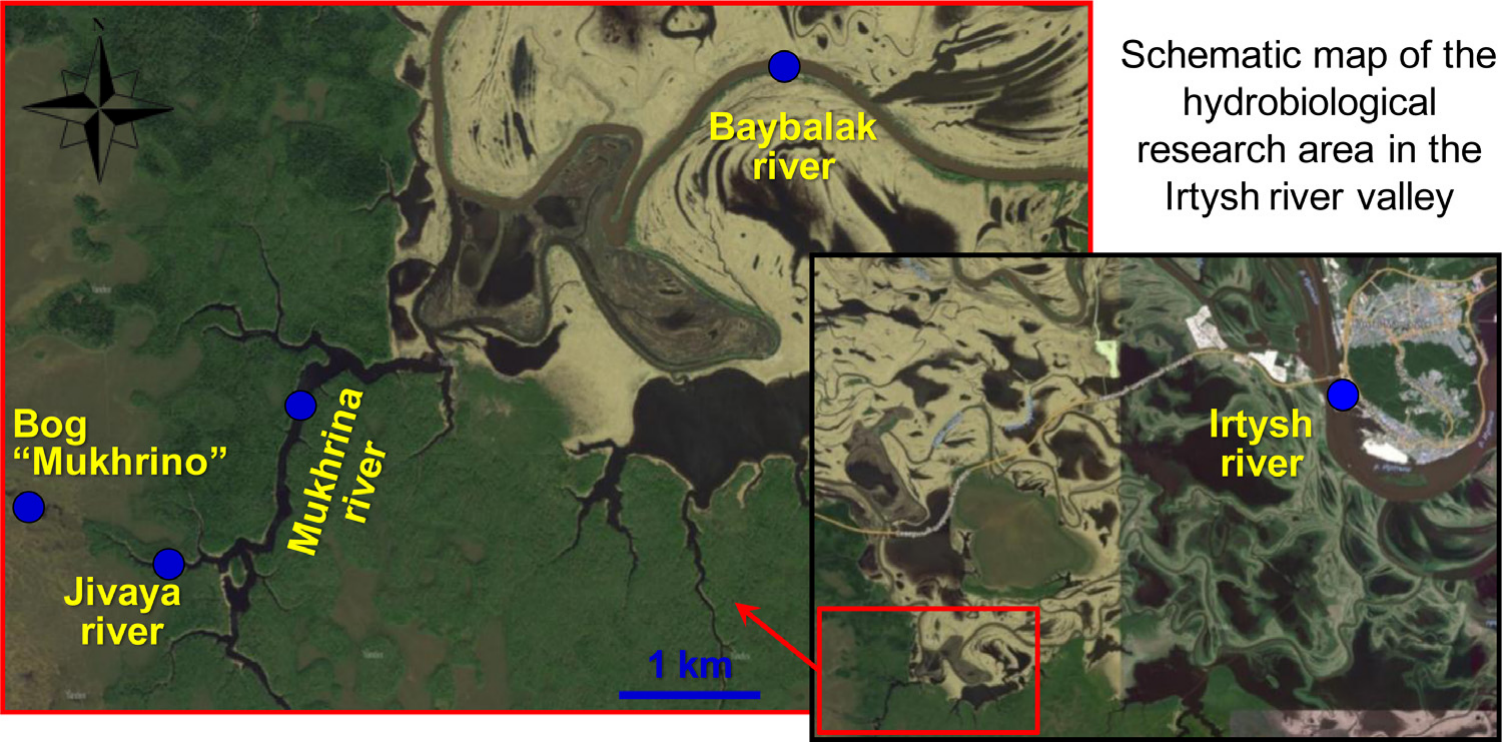
Schematic map of the sampling sites in the river valley of Irtysh, West Siberia. Blue dots denotes sampling sites.

In Fig. 1 show schematic map of the sampling sites at river valley of Irtysh, West Siberia

**Fig. 2:**
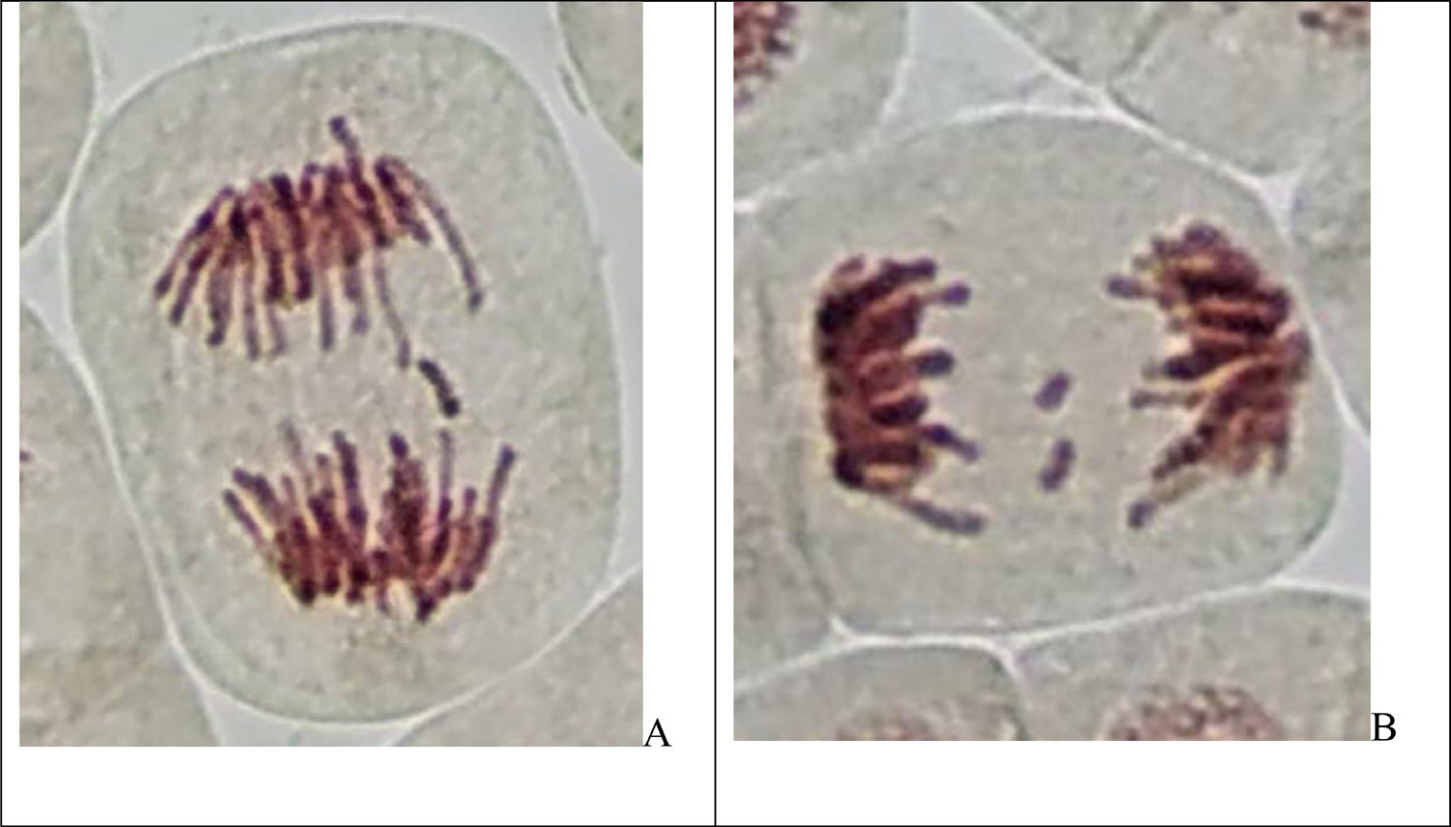

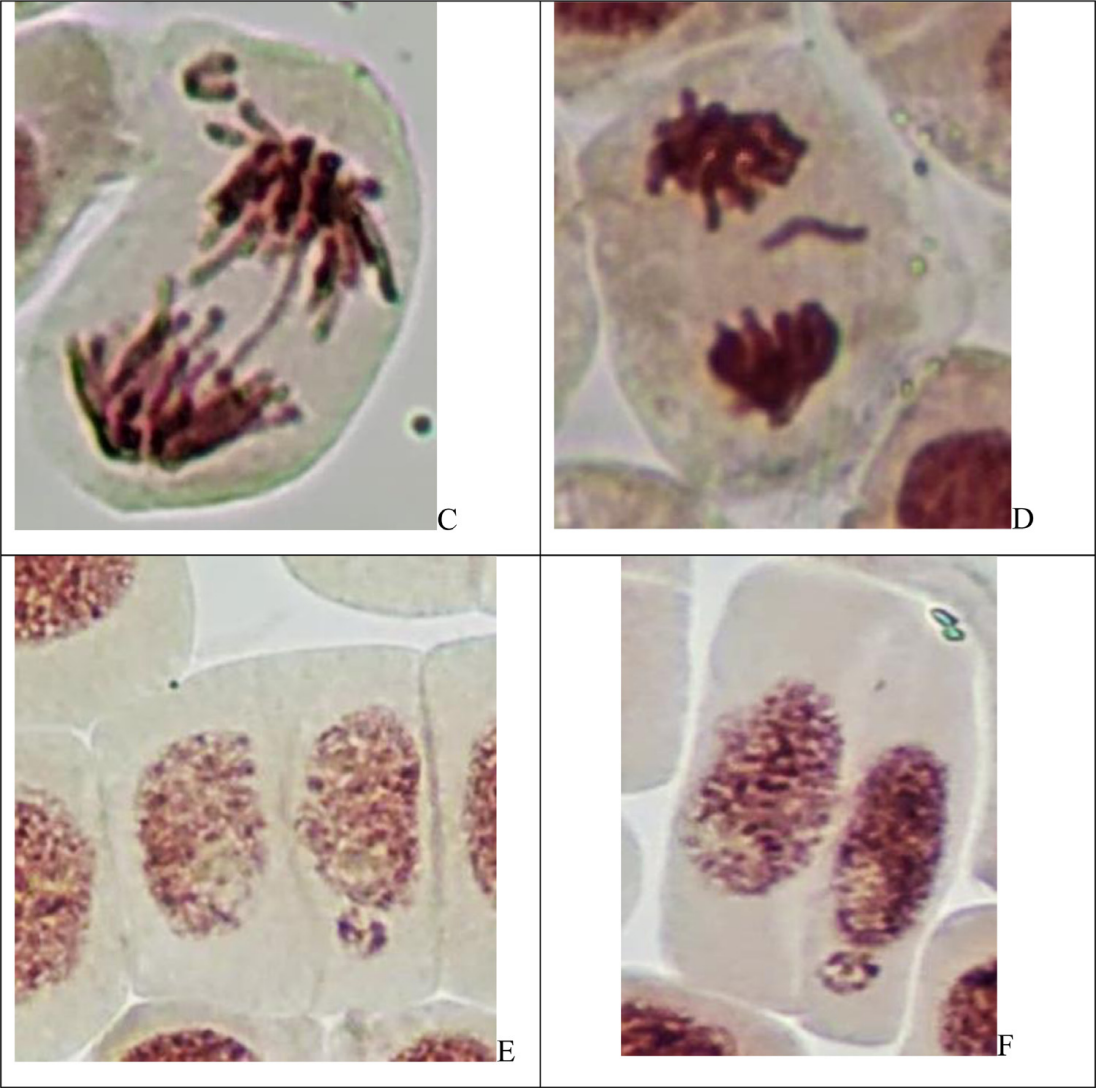
Microphotography of the cells in root tips of *Allium cepa L*. exposed to the natural waters from sampling sites. Types of abnormalities (A – single fragment, B – double fragment, C – chromosome bridge and fragment, D – chromosome loss, E and F – micronuclei).

In Fig. 2 shown photography of the cells with chromosomal and mitotic abnormalities, micronuclei in root tips of *Allium cepa L*.

**Fig. 3:**
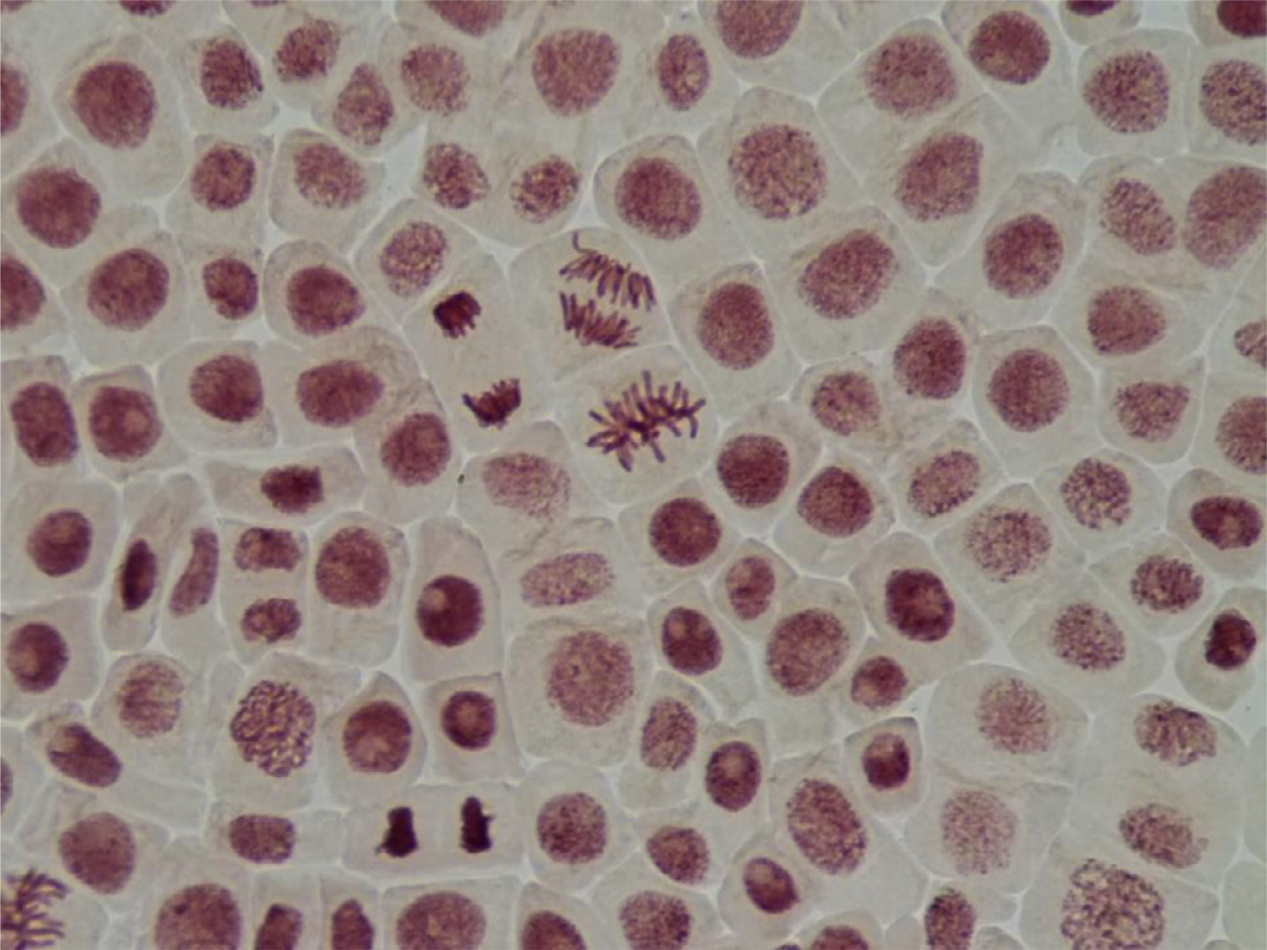
Microphotography of the cells in root tips of *Allium cepa L*. with different phases of mitosis and interphase cells.

In Fig. 3 shown microphotography of the cells in root tips of *Allium cepa L*. with different phases of mitosis and interphase cells.

## 2 Experimental Design, Materials and Methods

Our work presents the materials of a comprehensive hydroecological study (hydrology, hydrochemistry, genotoxicity) of various lakes and rivers of the left-bank Lower Irtysh valley, carried out in August 2021. The studies covered the lower reaches of the Irtysh River, small floodplain rivers (Baybalak, Mukhrina and Jivaya), as well as terrace bog lake massif Mukhrino.

The study area belongs to the left-bank subsystem of the Lower Irtysh valley, located 13 km above the confluence zone with the waters of the river Obi. The slopes of the above-floodplain terrace are heavily swampy and covered with mixed forest. The river valley is trapezoidal, 18 km wide. The floodplain is covered with meadow-shrub vegetation, has numerous channels, small rivers, lakes and oxbows.

Terrace bog ponds are small (<2-3 m2) accumulations of highly colored dystrophic waters formed in swamp depressions. The depth of the lakes are 30-40 cm, the water body is formed by dense thickets of sphagnum, sheuchzeria, watchtower, sedges, in some lakes - pemphigus.

Floodplain rivers are shallow, low-flowing, well-warmed streams with an increased content of labile organic matter in the water. The hydroecological feature of floodplain rivers are regular and prolonged (usually, from May to July-August) flooding during the spring-summer flood on the Irtysh River. This leads to the washout of hydrotoxicants from the floodplain and accumulation in floodplain rivers.

The Jivaya River is a small, low-flowing floodplain river. The river bed is not wide (10-25 m), with shallow depths (1-1.5 m) and dense thickets of aquatic plants. The banks of the river are soddy, heavily overgrown with hydrophilic vegetation. The floodplain rivers Baybalak and Mukhrina are deep, low-flowing streams, characterized by intensive processes of accumulation of alluvium. The width of the channel is 40-50 m. They are distinguished by high trophicity (in summer, a strong bloom of microalgae is regularly observed), which ensures high productivity of the aquatic community and effective feeding of numerous commercial fish species. Macrophytes are actively developing in shallow waters. The current velocity in the channel does not exceed 0.15 m/s.

The bed of the Lower Irtysh is wide (350 m), straight, slightly deformed. The river is fast-flowing (0.4-0.6 m/s), its waters are distinguished by increased turbidity (up to 130 mg/L) and high color, which is associated with a strong swampiness of the catchment area.

In the hydrobiological research area, the depth and temperature of the water were measured, the pH value, redox potential (ORP), electrical conductivity (Us) and the content of oxygen dissolved in water were determined with a portable multiparameter zonde YSI Pro. Under laboratory conditions, BOD_5_ was analyzed by titration (according to Winkler), and the concentration of biogenic components (nitrates and phosphates) and some typomorphic metal ions (iron, aluminum) were determined by spectrophotometry using a Hanna C-200 multiparameter analyzer. Water samples were taken from the lower reaches of the Irtysh River, small floodplain rivers Baybalak, Mukhrina and Jivaya, as well as from one of the terrace lake bog Mukhrino. Geography coordinates of sampling sites are shown in Table 3.

**Table 3:** Geography coordinates of sampling sites at rivel valley of Irtysh, West Siberia.

Sampling sites (names of water bodies)	Coordinates
Bog Mukhrino	60°53′29.95″N 68°41′6.04″E
Jivaya river	60°53′18.09″N 68°41′57.53″E
Mukhrina river	60°53′50.34″N 68°43′0.80″E
Baybalak river	60°54′54.75″N 68°47′22.05″E
Irtysh river	60°59′17.12″N 68°58′51.55″E

The Allium test was used to analyze genotoxic activity according to standard procedure [2,^3^,5]. The Allium test is recommended as standard in environmental monitoring and especially for aquatic ecosystem assessment [3,^4^,5]. When standardizing the method, it is reported that the data obtained in the Allium test can be used to assess genotoxicity not only for plants, but for eukaryotes in general, including humans [3]. Allium test data have a good correlation with other test systems: algae, fish, bacteria (Ames test), tests on animal cell cultures and human lymphocytes. Thus, the results obtained in the allium test are sufficient to make a quick and reliable assessment of the genotoxicity of various factors (including water assessment) in ecotoxicological studies [5]. The onion bulbs (*Allium cepa L*., 2n = 16) were of the Stuttgarten-Risen variety, average weight 25 g. In experiment used samples of natural water from 5 sampling sites of lower Irtysh river (West Siberia) – total 5 groups + 1 control group. For each group used 10 bulbs (60 for all groups in total). Bulbs of *A. cepa* were placed in small glass jars with their basal ends dipped in distilled water (control group) and in the water from sampling sites (experimental groups) and germinated at room temperature (24 ± 3°C) for 48 h. Then roots were fixed in Clarke's solution. For each group 10 slides prepared to analyse microscopic parameters. Aceto-orcein staining used. For each group analysed at least 5000 cells. Applied ana-telophase chromosomal aberration assay (to detect fragments and chromosome bridges) and micronuclei test (to detect small and large micronuclei in the interphase cells). Mitotic abnormalities were registered as laggard chromosomes and chromosome loss. Total abnormalities represent frequency of fragments, chromosome bridges, laggard chromosomes and chromosome loss. The mitotic index was calculated for each slide as a number of dividing cells (prophases, metaphases, anaphases, telophases) per at least 500 cells and also scored the proportions of mitotic phases (prophase index, metaphase index, anaphase index, telophase index). To analyse cells used light microscopy at 400-1000x.

## Ethics Statements

Not applicable. In the experiment we used plant test-object: *Allium cepa L.*

No any human subjects, animals or social media platforms data involved.

## Credit Author Statement

**Sergey Bolotov:** collecting water samples at sampling sites; writing – original draft; data calculation; **Dmitry Pesnya**: Allium test; raw data and data calculation; writing – original draft.

## Declaration of Competing Interest

The authors declare that they have no known competing financial interests or personal relationships that could have appeared to influence the work reported in this paper.
